# A Bias Compensation Method for Distributed Moving Source Localization Using TDOA and FDOA with Sensor Location Errors

**DOI:** 10.3390/s18113747

**Published:** 2018-11-02

**Authors:** Zhixin Liu, Rui Wang, Yongjun Zhao

**Affiliations:** 1 National Digital Switching System Engineering and Technological Research Center (NDSC), Zhengzhou 450002, China; liuzhixin54@sina.com (Z.L.); paper_zyj@sina.com (Y.Z.); 2 Institute of Surveying and Mapping, Information Engineering University; Zhengzhou 450002, China

**Keywords:** distributed localization, bias compensation, sensor location errors, time difference of arrival, frequency difference of arrival

## Abstract

Current bias compensation methods for distributed localization consider the time difference of arrival (TDOA) and frequency difference of arrival (FDOA) measurements noise, but ignore the negative influence by the sensor location uncertainties on source localization accuracy. Therefore, a new bias compensation method for distributed localization is proposed to improve the localization accuracy in this paper. This paper derives the theoretical bias of maximum likelihood estimation when the sensor location errors and positioning measurements noise both exist. Using the rough estimate result by MLE to subtract the theoretical bias can obtain a more accurate source location estimation. Theoretical analysis and simulation results indicate that the theoretical bias derived in this paper matches well with the actual bias in moderate noise level so that it can prove the correctness of the theoretical derivation. Furthermore, after bias compensation, the estimate accuracy of the proposed method achieves a certain improvement compared with existing methods.

## 1. Introduction

Estimation of the source location has been a subject of research for decades and continues to receive much interest in the signal processing research community [1,2,3], including radar [4], sonar [5], sensor network [6], wireless communication [2], etc. There are various common measurements employed to determine the source location, such as the time difference of arrival (TDOA), the frequency difference of arrival (FDOA), and numerous joint algorithms of multiple measurements. For simplicity, the TDOA has been extensively studied to improve estimation accuracy with a low computational complexity for solving source location which must lie in the intersection of the TDOA hyperbolic curves [7]. If there is relative motion between the source and sensors, the FDOA can be incorporated with the TDOA [8], which can significantly improve the source location accuracy and estimate the position and velocity of the source simultaneously [9]. Thus, we locate the moving source using TDOA and FDOA measurements in this paper.

**Figure 1:**
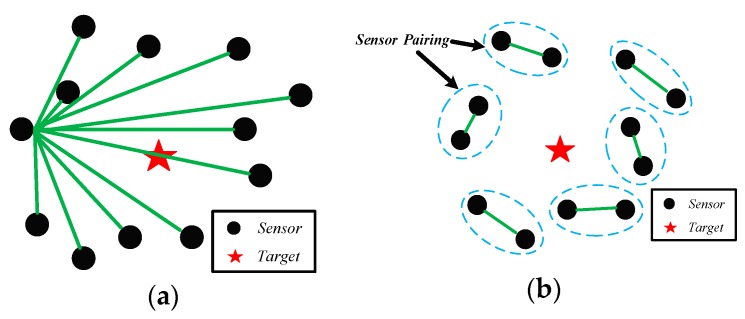
Sensor pairing. (**a**) Centralized Localization; (**b**) Distributed localization.

Based on the source localization using TDOA and FDOA measurements, the two types of sensor pairing structure are presented [10,11,12,13], which are shown in Figure 1. Most of source localization method adopt the centralized localization structure (Figure 1a), such as iteration-based method [14,15,16], two-step weighted least squares (TS-WLS) [9,10], total least-squares (TLS) technique [17], the semi-definite relaxation localization method [18], the multidimensional scaling (MDS) method [19] and so on. However, for joint TDOA- and FDOA-based methods, time-synchronization and frequency-locking are typically required [20], which might be difficult to achieve and increase the system complexity [21]. If this system fails to achieve precise synchronization between all the sensors, it may not obtain correct measurements from received signals so that the estimated accuracy cannot be ensured [22]. In addition, according to refs. [23,24,25], some centralized sensors may not transmit the data to their single reference sensor due to their limited communication range and system power. Moreover, the single reference sensor needs to save and use to estimates parameters from all the data, which will produce high computational costs and may also cause a large processing delay [25]. These requirements would have significant influence on the size, weight, and power of that sensor [26].

To circumvent the drawbacks of centralized localization system, the distributed localization is highly desirable [11,12,13,27]. As seen in the Figure 1b, sensor pairs is combined in a decentralized way to estimate the corresponding TDOAs and FDOAs. There are several advantages using this localization structure. Firstly, because of no common reference sensor, the sensors of distributed structure only need to transmit their original data to another sensor of each group. Secondly, due to the pairing structure of distributed localization, it lies in the lower requirement for synchronization [11]. We only need to achieve precise synchronization between two sensors in a group rather than all sensors, which is easy to realize in practice. Overall, this structure could improve robustness, and save bandwidth of the communication network, which can reduce the difficulty of success in engineering [25,27]. Therefore, we aim at distributed structure localization algorithm based on TDOA and FDOA in this paper.

The estimated accuracy is usually not guaranteed by using classical maximum likelihood estimation (MLE) methods because of the nonlinearity in TDOA and FDOA localization problem. Thus, the MSE consists of the variance and the bias square [28]. Increasing the observation period can only decrease the location variance but the bias cannot be ignored. For example, the ultra-wideband (UWB) localization technology [29] use the averaging to reduce estimated variance but is useless to decrease the estimated bias, which cause negative influence on location accuracy [28,30].

Therefore, the question of how to remove the bias from the estimation of source position and velocity is a focus of the research. In the last decade, many bias compensation algorithms were proposed [11,28,29,30,31,32]. As for stationary emitters, Rui L verified that the location bias has great influence on location accuracy [28,30]. In order to avoid it, Hao put forward a bias reduction method for passive source localization using TDOA and gain ratios of arrival (GROA) [32]. As for moving sources, Chan proposed a new bias reduction algorithm using new constraints based on TDOA and FDOA [31]. However, these algorithms can only reduce the bias to the same degree of MLE, which is still high for an estimation result, and do not consider the sensor location uncertainties which are very sensitive to the source location accuracy [10]. As is well known, the position and velocity of sensors may not be obtained accurately in practice when using moving sensors [10]. Therefore, the sensor location uncertainties need to be taken into consideration in practical environment. Therefore, a new bias compensation method based on MLE for distributed source localization using TDOA and FDOA with sensor location errors is presented in this paper.

We study the bias of the MLE for source location, because the MLE is asymptotically efficient and regarded as a benchmark for performance evaluation [33]. The bias of the MLE of a general estimation problem has been investigated in the mathematical and statistical literature [33]. The proposed method extends the method in References [1,11], and derives the theoretical bias of MLE when the localization model has sensor location errors. The bias expression is closed-form with low computationally cost and the source location accuracy has a certain increase after bias compensation.

The paper is organized as follows. Section 2 formulates the problem of distributed source localization using TDOA and FDOA measurements with receiver location errors. Section 3 gives a detailed derivation of the proposed method. Section 4 derives the Cramér-Rao lower bound (CRLB) for distributed localization. Section 5 presents simulation to support the theoretical development of the proposed method. Finally, a brief conclusion is given in Section 6.

## 2. Measurement Model

Due to the no common reference sensor of the distributed localization, the model of distributed localization is different from centralized model. In this paper, we consider the three-dimensional (3-D) scenario, thus the distributed structure requires at least three pairs of sensors (i.e. *M* = 6) to produce the several groups of TDOA and FDOA measurements and *M* must be an even number. Each sensor position and velocity can be defined as $s_{i}={\left[x_{i},y_{i},z_{i}\right]}^{T}$ and ${\dot{s}}_{i}={\left[{\dot{x}}_{i},{\dot{y}}_{i},{\dot{z}}_{i}\right]}^{T}$ (i=1,2,…,M) respectively, which is applied to determine the source position $u={\left[x,y,z\right]}^{T}$ and velocity $\dot{u}={\left[\dot{x},\dot{y},\dot{z}\right]}^{T}$. We will use the notation ${\left(*\right)}^{o}$ and (∗) to distinguish between the true value and noisy value. In practice, the true value of the sensor position $s_{i}^{o}={\left[x_{i}^{o},y_{i}^{o},z_{i}^{o}\right]}^{T}$ and velocity ${\dot{s}}_{i}^{o}={\left[{\dot{x}}_{i}^{o},{\dot{y}}_{i}^{o},{\dot{z}}_{i}^{o}\right]}^{T}$ are unknown to an estimator, thus we only use the noisy value $s_{i}$ and ${\dot{s}}_{i}$ to derive the localization method [10].

(1) $$\Delta \beta =n_{\beta }=\beta -\beta^{o}={\left[\Delta s^{T},\Delta {\dot{s}}^{T}\right]}^{T}$$

where $\beta ={\left[s^{T},{\dot{s}}^{T}\right]}^{T}$, $\Delta s_{i}=s_{i}-s_{i}^{o}$, $\Delta {\dot{s}}_{i}={\dot{s}}_{i}-{\dot{s}}_{i}^{o}$, $\Delta s={\left[\Delta s_{1}^{T},\Delta s_{2}^{T},\ldots ,\Delta s_{M}^{T}\right]}^{T}$, and $\Delta \dot{s}={\left[\Delta {\dot{s}}_{1}^{T},\Delta {\dot{s}}_{2}^{T},\ldots ,\Delta {\dot{s}}_{M}^{T}\right]}^{T}$. In this paper, Δβ is assumed to follows zero-mean Gaussian distribution with covariance matrix

(2) $$E\left[\Delta \beta \Delta \beta^{T}\right]=Q_{\beta }$$

The distance between the source and sensor *i*th is

(3) $$r_{i}^{o}=\left\|u^{o}-s_{i}^{o}\right\|=\sqrt{{\left(u^{o}-s_{i}^{o}\right)}^{T}\left(u^{o}-s_{i}^{o}\right)}$$

where ‖⋅‖ denotes the Euclidean norm. For *M* sensors, there are a total number of M/2 sensor pairs and TDOA/FDOA measurements. Let

(4) Σ={{2i,2i−1}|1≤i≤M/2}

which denotes the set of all sensor pairs. Without loss of generality, the sensor $s_{2i-1}$ be the reference sensor, which is the first sensor of each group. In the system, the TDOAs can be defined as range difference, and the range difference between the 2*i*th sensor and the sensor (2*i*−1)th is

(5) $$r_{2i,2i-1}^{o}=c\tau_{2i,2i-1}^{o}=r_{2i}^{o}-r_{2i-1}^{o}$$

where c is the propagation speed, $\tau_{2i,2i-1}^{o}$ is the true TDOA value between pair 2*i*th and (2*i*-1)th sensor, $r_{2i,2i-1}^{o}$ is the true range difference, and i=1,2,…,M/2. Note that (5) is nonlinear with respect to u, the M/2 curves in (5) give the source location estimate. 

Due to the moving source, the FDOA measurements can be used to improve the accuracy of source location estimate and obtain the source position and velocity simultaneously. Similarly, the FDOAs can be converted to the range difference rate. By taking the time derivative of (5), the true range rate ${\dot{r}}_{i}^{o}$ of the *i*th sensor is defined as

(6) $${\dot{r}}_{i}^{o}=\frac{{\left(u^{o}-s_{i}^{o}\right)}^{T}\left({\dot{u}}^{o}-{\dot{s}}_{i}^{o}\right)}{r_{i}^{o}}$$

Similarly, the range difference rate between the 2*i*th sensor and the sensor (2*i*−1)th is

(7) $${\dot{r}}_{2i,2i-1}^{o}={\dot{r}}_{2i}^{o}-{\dot{r}}_{2i-1}^{o}$$

for i=1,2,…,M/2. (5) and (7) are a set of TDOA and FDOA nonlinear equation, and it is not easy to obtain the source location u and $\dot{u}$ by solving them. Moreover, we cannot obtain the true values of TDOA and FDOA in practice. So, we let $\alpha ={\left[r^{T},{\dot{r}}^{T}\right]}^{T}$, where $r={\left[r_{2,1},r_{4,3},\ldots ,r_{M,M-1}\right]}^{T}$ and $\dot{r}={\left[{\dot{r}}_{2,1},{\dot{r}}_{4,3},\ldots ,{\dot{r}}_{M,M-1}\right]}^{T}$ represent the TDOA and FDOA measurements vector, then the vector of the measurements error is

(8) $$\Delta \alpha =n_{\alpha }={\left[\Delta r^{T},\Delta {\dot{r}}^{T}\right]}^{T}$$

where $\Delta r=r-r^{o}$, $\Delta r={\left[\Delta r_{2,1},\Delta r_{4,3},\ldots ,\Delta r_{M,M-1}\right]}^{T}$, $\Delta \dot{r}=\dot{r}-{\dot{r}}^{o}$, and $\Delta \dot{r}={\left[\Delta {\dot{r}}_{2,1},\Delta {\dot{r}}_{4,3},\ldots ,\Delta {\dot{r}}_{M,M-1}\right]}^{T}$. Similarly, we assume that Δα obeys zero-mean Gaussian distribution with covariance matrix

(9) $$E\left[\Delta \alpha \Delta \alpha^{T}\right]=Q_{\alpha }$$

The two types of noises, which are Δα and Δβ, are independent of each other and we assume that they are uncorrelated at different time instants. In next section, we analyze the theoretical bias of the MLE and derive its expression using the two types of positioning measurements with the sensor location errors, which can efficiently reduce the bias of estimated result and obtain more accurate source position u and velocity $\dot{u}$. In this paper, we use the 0, O, and I to denote the zero vector, zero matrix, and unit matrix, respectively. Their dimensions are marked in the lower right corner of themselves.

## 3. The Proposed Method

Although it is well known that the variance of the MLE can achieve the CRLB, the bias of the MLE still exists and has a negative influence on estimation accuracy. Therefore, in order to remove the bias of estimate result and improve the precision of the source location, in this section, we derive the theoretical bias of the MLE based on TDOA and FDOA measurements with sensor location errors. According to Section 2, the two types of positioning measurements noise Δα and sensor location errors noise Δβ both obey the Gaussian distribution with zero means which are independent of each other [7,9,10,18]. It should be explained that these types of noises in practical localization environments may not follow these assumptions in practical localization environments. We will do some effort in other noise environment in our further study, but these types of noises are only used to as an example to derive the proposed method in this paper. Thus, according to the assumption mentioned above, the log of the joint probability density function of α and β parameterized on φ is [10]

(10) $$\begin{array}{ll} \ln f\left(\alpha ,\beta ,\varphi \right) & =\ln f\left(\alpha ,\varphi \right)+\ln f\left(\beta ,\varphi \right)\\ & =K_{1}-\frac{1}{2}{\left[\alpha -\alpha^{o}\right]}^{T}Q_{\alpha }{}^{-1}\left[\alpha -\alpha^{o}\right]-\frac{1}{2}{\left[\beta -\beta^{o}\right]}^{T}Q_{\beta }{}^{-1}\left[\beta -\beta^{o}\right] \end{array}$$

where $K_{1}=-1/2\ln\left({\left(2\pi \right)}^{M}\left|Q_{\alpha }\right|\right)-1/2\ln\left({\left(2\pi \right)}^{6M}\left|Q_{\beta }\right|\right)$, $\varphi ={\left[\theta^{T},\beta^{T}\right]}^{T}$, $\theta ={\left[u^{T},{\dot{u}}^{T}\right]}^{T}$. Since the noise follows zero mean Gaussian distribution and only the θ is of interest to us, so the MLE solution $\hat{\theta }$ is

(11) $$\hat{\theta }=\arg\;\max\left(I\right)$$

where the cost function *I* of ML estimation can be formulated by

(12) $$I≜-\frac{1}{2}{\left[\alpha -\alpha^{o}\right]}^{T}Q_{\alpha }{}^{-1}\left[\alpha -\alpha^{o}\right]-\frac{1}{2}{\left[\beta -\beta^{o}\right]}^{T}Q_{\beta }{}^{-1}\left[\beta -\beta^{o}\right]$$

We use $P\left(\hat{\varphi }\right)$ to stand for the gradient of the cost function I with respect to φ, $\hat{\varphi }$ satisfies the equation

(13) $$P\left(\hat{\varphi }\right)={\frac{\partial I}{\partial \varphi }|}_{\hat{\varphi }}=0_{\left(6M+6\right)\times 1}$$

Then, we apply the second-order Taylor-series approximation to $P\left(\hat{\varphi }\right)$ around $\varphi^{o}$, which can be represented as

(14) $$P\left(\hat{\varphi }\right)={\frac{\partial I}{\partial \varphi }|}_{\hat{\varphi }}\approx H^{\prime }+H^{\prime \prime }\left(\hat{\varphi }-\varphi^{o}\right)+g\left(\varphi^{o}\right)=0_{\left(6M+6\right)\times 1}$$

where

(15) $$\begin{array}{l} H^{\prime }={\frac{\partial I}{\partial \varphi }|}_{\varphi =\varphi^{o}},\;H^{\prime \prime }={\frac{\partial^{2}I}{\partial \varphi \partial \varphi^{T}}|}_{\varphi =\varphi^{o}}\\ {H^{\prime \prime \prime }}_{l}={\frac{\partial }{\partial \varphi_{l}}\left(\frac{\partial^{2}I}{\partial \varphi \partial \varphi^{T}}\right)|}_{\varphi =\varphi^{o}}\;l=1,2,\ldots ,6M+6\\ g\left(\varphi^{o}\right)=\frac{1}{2}\left[\begin{array}{c} tr\left({H^{\prime \prime \prime }}_{1}\times \left[\hat{\varphi }-\varphi^{o}\right]{\left[\hat{\varphi }-\varphi^{o}\right]}^{T}\right)\\ tr\left({H^{\prime \prime \prime }}_{2}\times \left[\hat{\varphi }-\varphi^{o}\right]{\left[\hat{\varphi }-\varphi^{o}\right]}^{T}\right)\\ \vdots \\ tr\left({H^{\prime \prime \prime }}_{6M+6}\times \left[\hat{\varphi }-\varphi^{o}\right]{\left[\hat{\varphi }-\varphi^{o}\right]}^{T}\right) \end{array}\right] \end{array}$$

and tr(∗) represents the trace operation. 

Based on (14), we notice that there is no need to solve $\hat{\varphi }$ and the bias is directly given as the expectation of $\hat{\varphi }-\varphi^{o}$. Thus, we rearrange (14) and obtain the theoretical bias

(16) $$b=E\left[\hat{\varphi }-\varphi^{o}\right]=E\left[-{\left(H^{\prime \prime }\right)}^{-1}H^{\prime }\right]+E\left[-{\left(H^{\prime \prime }\right)}^{-1}g\left(\varphi^{o}\right)\right]$$

The details of (14) is specifically shown as

(17) $$\begin{array}{l} H^{\prime }={\frac{\partial I}{\partial \varphi }|}_{\varphi =\varphi^{o}}=C_{1}+C_{2}\\ H^{\prime \prime }={\frac{\partial^{2}I}{\partial \varphi \partial \varphi^{T}}|}_{\varphi =\varphi^{o}}=\left(B_{1}-A_{1}\right)+\left(B_{2}-A_{2}\right) \end{array}$$

where

(18) $$\begin{array}{l} A_{1}={\frac{\partial^{T}\alpha }{\partial \varphi }Q_{\alpha }^{-1}\frac{\partial \alpha }{\partial \varphi^{T}}|}_{\varphi =\varphi^{o}}\\ A_{2}={\frac{\partial^{T}\beta }{\partial \varphi }Q_{\beta }^{-1}\frac{\partial \beta }{\partial \varphi^{T}}|}_{\varphi =\varphi^{o}}\\ B_{1}={\sum_{j=1}^{M}\sum_{i=1}^{M}q_{\alpha }{}_{ij}n_{\alpha }{}_{i}\frac{\partial^{2}\alpha_{j}}{\partial \varphi \partial \varphi^{T}}|}_{\varphi =\varphi^{o}}\\ B_{2}={\sum_{j=1}^{2M}\sum_{i=1}^{2M}q_{\beta }{}_{ij}n_{\beta }{}_{i}\frac{\partial^{2}\beta_{j}}{\partial \varphi \partial \varphi^{T}}|}_{\varphi =\varphi^{o}}\\ C_{1}={\frac{\partial^{T}\alpha }{\partial \varphi }Q_{\alpha }^{-1}n_{\alpha }|}_{\varphi =\varphi^{o}}\\ C_{2}={\frac{\partial^{T}\beta }{\partial \varphi }Q_{\beta }^{-1}n_{\beta }|}_{\varphi =\varphi^{o}} \end{array}$$

and $q_{\alpha }{}_{ij}$ and $q_{\beta }{}_{ij}$ are the element of $Q_{\alpha }^{-1}$ and $Q_{\beta }^{-1}$. For specifically, we let $A=A_{1}+A_{2}$ in the following. 

### 3.1. The Derivation of The First Term of (16)

This section gives a detailed derivation of the first term of (16) $E[-{\left(H^{\prime \prime }\right)}^{-1}H^{\prime }]$. According to the (17) and (18), the first term of (16) can be approximated as

(19) $$\begin{array}{ll} E\left[-{\left(H^{\prime \prime }\right)}^{-1}H^{\prime }\right] & =E\left[{\left(A-\left(B_{1}+B_{2}\right)\right)}^{-1}\left(C_{1}+C_{2}\right)\right]\\ & \approx E\left[A^{-1}\left(C_{1}+C_{2}\right)\right]+E\left[A^{-1}\left(B_{1}+B_{2}\right)A^{-1}\left(C_{1}+C_{2}\right)\right]\\ & =E\left[A^{-1}\left(C_{1}+C_{2}\right)\right]+E\left[A^{-1}B_{1}A^{-1}C_{2}\right]\\ & +E\left[A^{-1}B_{2}A^{-1}C_{1}\right]+E\left[A^{-1}\left(B_{1}A^{-1}C_{1}+B_{2}A^{-1}C_{2}\right)\right] \end{array}$$

Note that the $A^{-1}$ does not contain noise and it is independent of the measurement noise and the sensor location noise, then we have

(20) $$\begin{array}{l} E\left[A^{-1}\left(C_{1}+C_{2}\right)\right]=E\left[A^{-1}C_{1}\right]+E\left[A^{-1}C_{2}\right]=0_{\left(6M+6\right)\times 1}\\ E\left[A^{-1}B_{1}A^{-1}C_{2}\right]=0_{\left(6M+6\right)\times 1}\\ E\left[A^{-1}B_{2}A^{-1}C_{1}\right]=0_{\left(6M+6\right)\times 1} \end{array}$$

So, the (19) can be simplified as

(21) $$\begin{array}{cc} E\left[-{\left(H^{\prime \prime }\right)}^{-1}H^{\prime }\right] & \approx E\left[A^{-1}\left(B_{1}+B_{2}\right)A^{-1}\left(C_{1}+C_{2}\right)\right]\\ & =E\left[A^{-1}\left(B_{1}A^{-1}C_{1}+B_{2}A^{-1}C_{2}\right)\right] \end{array}$$

Substituted the definition of $B_{1},B_{2},C_{1},C_{2}$ from (18) and $E\left[n_{\alpha }{}_{i}n_{\alpha }\right]=Q_{\alpha }e_{\alpha }{}_{i}$,$E\left[n_{\beta }{}_{i}n_{\beta }\right]=Q_{\beta }e_{\beta }{}_{i}$, the first term of (16) is

(22) $$\begin{array}{cc} E\left[-{\left(H^{\prime \prime }\right)}^{-1}H^{\prime }\right] & \approx A^{-1}\sum_{i=1}^{M}P_{\alpha }{}_{i}A^{-1}\left(\frac{\partial^{T}\alpha }{\partial \varphi }\right)Q_{\alpha }^{-1}\cdot E\left[n_{\alpha }{}_{i}n_{\alpha }\right]+A^{-1}\sum_{i=1}^{2M}P_{\beta }{}_{i}A^{-1}\left(\frac{\partial^{T}\beta }{\partial \varphi }\right)Q_{\beta }^{-1}\cdot E\left[n_{\beta }{}_{i}n_{\beta }\right]\\ & ={A^{-1}\sum_{i=1}^{M}P_{\alpha }{}_{i}A^{-1}\left(\frac{\partial^{T}\alpha }{\partial \varphi }\right)e_{\alpha }{}_{i}+A^{-1}\sum_{i=1}^{2M}P_{\beta }{}_{i}A^{-1}\left(\frac{\partial^{T}\beta }{\partial \varphi }\right)e_{\beta }{}_{i}|}_{\varphi =\varphi^{o}} \end{array}$$

where $e_{\alpha }{}_{i}$ and $e_{\beta }{}_{i}$ are M×1 and 6M×1 vector respectively which are defined as

(23) $$\begin{array}{l} e_{\alpha }{}_{i}={\left[\overset{M}{\overset{︷}{\underset{i-1}{\underset{︸}{\begin{array}{ccc} 0 & \cdots & 0 \end{array}}}\begin{array}{ccc} \;\underset{i}{1}\;0 & \cdots & 0 \end{array}}}\right]}^{T}\\ e_{\beta }{}_{i}={\left[\overset{6M}{\overset{︷}{\underset{i-1}{\underset{︸}{\begin{array}{ccc} 0 & \cdots & 0 \end{array}}}\begin{array}{ccc} \underset{i}{1}\;0 & \cdots & 0 \end{array}}}\right]}^{T} \end{array}$$

and the $P_{\alpha }{}_{i}$ and $P_{\beta }{}_{i}$ are expressed as

(24) $$\begin{array}{l} P_{\alpha }{}_{i}={\sum_{j=1}^{M}q_{\alpha }{}_{ij}\frac{\partial^{2}\alpha_{j}}{\partial \varphi \partial \varphi^{T}}|}_{\varphi =\varphi^{o}}\\ P_{\beta }{}_{i}={\sum_{j=1}^{2M}q_{\beta }{}_{ij}\frac{\partial^{2}\beta_{j}}{\partial \varphi \partial \varphi^{T}}|}_{\varphi =\varphi^{o}} \end{array}$$

### 3.2. The Derivation of The Second Term of (16)

The second term of (16) $E\left[-{\left(H^{\prime \prime }\right)}^{-1}g\left(\varphi^{o}\right)\right]$ is quite complex to derive and we will do some approximation processing [1]. According to refs. [1,28,30,31], because the $B_{1}$ and $B_{2}$ contain the first-order noise term and $g(\varphi^{o})$ includes the second-order noise term, multiplying them together can produce the high-order (third-order) noise terms which are lower enough to ignore when the method estimates at low noise level. And high-order noise terms are significantly small than the low-order term, which is reasonable to ignore. Thus, from (17), we have $H^{\prime \prime }\approx -A$ and the second bias component is approximately expressed as

(25) $$E\left[-{\left(H^{\prime \prime }\right)}^{-1}g\left(\varphi^{o}\right)\right]\approx A^{-1}z$$

where

(26) $$z≜E\left[g\left(\varphi^{o}\right)\right]\approx \frac{1}{2}{\left[\begin{array}{c} tr\left(E\left[{H^{\prime \prime \prime }}_{1}\right]\times CRLB\left(\varphi^{o}\right)\right)\\ tr\left(E\left[{H^{\prime \prime \prime }}_{2}\right]\times CRLB\left(\varphi^{o}\right)\right)\\ \vdots \\ tr\left(E\left[{H^{\prime \prime \prime }}_{6M+6}\right]\times CRLB\left(\varphi^{o}\right)\right) \end{array}\right]}_{\left(6M+6\right)\times 1}$$

The $CRLB(\varphi^{o})$ is the CRLB of the true value $\varphi^{o}$ whose bias is neglected. In fact, the MLE is also efficient after valid approximation. In addition, according to the Appendix D, the $\partial^{2}\beta_{j}/\partial \varphi \partial \varphi^{T}$ is a 6M+6 zero square matrix, so the $E\left[{H^{\prime \prime \prime }}_{l}\right]$ can be approximated by

(27) $$E\left[{H^{\prime \prime \prime }}_{l}\right]=\sum_{i=1}^{M}\left[h_{\alpha i}^{T}e_{l}P_{\alpha i}+P_{\alpha i}e_{l}h_{\alpha i}^{T}+h_{\alpha i}e_{l}^{T}P_{\alpha i}^{T}\right],\;\left(l=1,2,\ldots ,6M+6\right)$$

where

(28) $$h_{\alpha i}={\sum_{j=1}^{M}q_{\alpha ij}\frac{\partial^{T}\alpha_{j}}{\partial \varphi }|}_{\varphi =\varphi^{o}}$$

and $e_{l}$ is (6M+6)×1 vector which is defined as

(29) $$e_{l}={\left[\overset{6M+6}{\overset{︷}{\underset{l-1}{\underset{︸}{\begin{array}{ccc} 0 & \cdots & 0 \end{array}}}\begin{array}{ccc} \;\underset{l}{1}\;0 & \cdots & 0 \end{array}}}\right]}^{T}$$

### 3.3. The Algebraic Expression of Bias

Based on Section 3.1 and Section 3.2, the bias is equal to

(30) $$b=E\left[\hat{\varphi }-\varphi^{o}\right]={A^{-1}\left(\sum_{i=1}^{M}P_{\alpha i}A^{-1}\left(\frac{\partial^{T}\alpha }{\partial \varphi }\right)e_{\alpha i}+z\right)+A^{-1}\sum_{i=1}^{2M}P_{\beta i}A^{-1}\left(\frac{\partial^{T}\beta }{\partial \varphi }\right)e_{\beta i}|}_{\varphi =\varphi^{o}}$$

According to the Appendix D, the $P_{\beta i}$ is a 6M+6 zero square matrix. Thus the final component of the bias can be obtained

(31) $$b=E\left[\hat{\varphi }-\varphi^{o}\right]={A^{-1}\left(\sum_{i=1}^{M}P_{\alpha i}A^{-1}\left(\frac{\partial^{T}\alpha }{\partial \varphi }\right)e_{\alpha i}+z\right)|}_{\varphi =\varphi^{o}}$$

Equation (31) is the theoretical bias of the MLE which is the closed-form with reasonable computational complexity $O(M^{2})$ in processing. The details on the evaluation of the derivatives for α and β is given in Appendix A, Appendix B, Appendix C and Appendix D. The bias of the MLE is accurately predicted by using (31), hence the current source position and velocity after bias compensation is given by

(32) $$\tilde{\varphi }=\hat{\varphi }-b$$

$\tilde{\varphi }$ can approximately be regarded as an unbiased estimator of $\varphi^{o}$ with covariance matrix $CRLB(\varphi^{o})$ and only the $\tilde{\theta }$ of $\tilde{\varphi }$ is of interest for us. In practice, due to the unknown source location, we use MLE estimated results instead of the true value in the bias expression (31). We will present the computer simulations to corroborate our theoretical development and to compare the relative location accuracy for different methods in Section 5.

## 4. CRLB for Distributed Localization with Receiver Location Errors

The CRLB is the lowest possible variance that an unbiased estimator can achieve which is often regarded as the benchmark of estimation performance [9]. This section derives the CRLB of $\varphi^{o}$ in distributed passive sensor localization system in the presence of sensor location errors under the Gaussian noise model for the first time. 

According to (10), assuming J is the (6M+6)×(6M+6) Fisher Information Matrix (FIM) [34], which is defined as

(33) $$J=E{\left[-\left(\frac{\partial^{2}\ln f\left(\alpha ,\beta ,\varphi \right)}{\partial \varphi \partial \varphi^{T}}\right)\right]|}_{\varphi =\varphi^{o}}$$

The CRLB of $\varphi^{o}$ is equal to the inverse of the Fisher matrix [9,10] defined as

(34) $$\mathrm{CRLB}\left(\varphi^{o}\right)=J^{-1}=-E{\left[{\left(\frac{\partial^{2}\ln f\left(\alpha ,\beta ,\varphi \right)}{\partial \varphi \partial \varphi^{T}}\right)|}_{\varphi =\varphi^{o}}\right]}_{\left(6M+6\right)\times \left(6M+6\right)}^{-1}$$

Note (34) that we only focus on the upper left 6×6 submatrix of (34), which is the CRLB [10] of $\theta^{o}={\left[u^{o}{}^{T},{\dot{u}}^{o}{}^{T}\right]}^{T}$. In order to express simplicity, we convert (34) into submatrix form shown as

(35) $$CRLB\left(\varphi^{o}\right)={\left[\begin{array}{cc} R_{1} & R_{2}\\ R_{2}^{T} & R_{3} \end{array}\right]}^{-1}$$

where

(36) $$\begin{array}{l} R_{1}=E{\left[\left(\frac{\partial^{2}\ln f\left(\alpha ,\beta ,\varphi \right)}{\partial \theta \partial \theta^{T}}\right)\right]|}_{\varphi =\varphi^{o}}={\frac{\partial^{T}\alpha }{\partial \theta }Q_{\alpha }^{-1}\frac{\partial \alpha }{\partial \theta^{T}}|}_{\varphi =\varphi^{o}}\\ R_{2}=E{\left[\left(\frac{\partial^{2}\ln f\left(\alpha ,\beta ,\varphi \right)}{\partial \theta \partial \beta^{T}}\right)\right]|}_{\varphi =\varphi^{o}}={\frac{\partial^{T}\alpha }{\partial \theta }Q_{\alpha }^{-1}\frac{\partial \alpha }{\beta^{T}}|}_{\varphi =\varphi^{o}}\\ R_{3}=E{\left[\left(\frac{\partial^{2}\ln f\left(\alpha ,\beta ,\varphi \right)}{\partial \beta \partial \beta^{T}}\right)\right]|}_{\varphi =\varphi^{o}}={\frac{\partial^{T}\alpha }{\partial \beta }Q_{\alpha }^{-1}\frac{\partial \alpha }{\partial \beta^{T}}+Q_{\beta }^{-1}|}_{\varphi =\varphi^{o}} \end{array}$$

The partial derivatives $\partial \alpha /\partial \varphi^{T}$ and $\partial \beta /\partial \varphi^{T}$ are given in Appendix A and Appendix B. According to the partitioned matrix inversion formula in [34], the (35) can be rewritten as

(37) $$CRLB\left(\varphi^{o}\right)=R_{1}^{-1}+R_{1}^{-1}R_{2}{\left(R_{3}-R_{2}^{T}R_{1}^{-1}R_{2}\right)}^{-1}R_{2}^{T}R_{1}^{-1}$$

Note that $R_{1}^{-1}$ is the CRLB in ref. [11] with no sensor location errors. Since the $\left(R_{3}-R_{2}^{T}R_{1}^{-1}R_{2}\right)$ is the positive definite matrix, the second term in (37) stands for the increase in CRLB after adding the sensor location errors [10]. So, the source localization algorithm is necessary to consider the sensor location errors, otherwise it could cause a serious impact on the estimation precision. In the next section, we compare the CRLB derived in this section with the CRLB without sensor location error in [11], which can indicate the relationship between the estimation performance and sensor location errors.

## 5. Numerical Simulations

**Figure 2:**
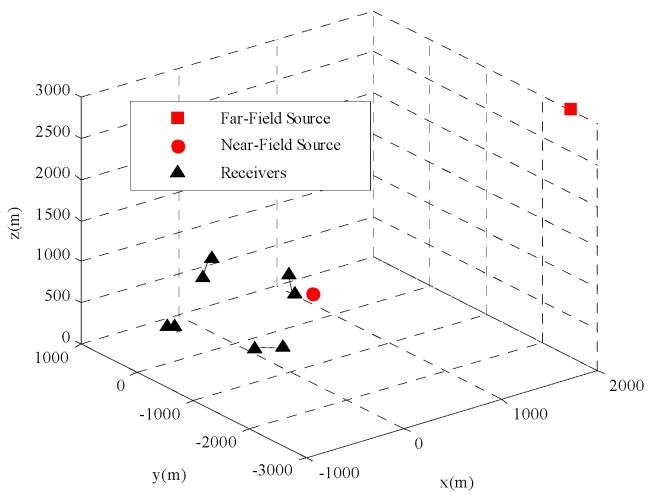
Localization geometry for simulation.

This section presents four sets of Monte Carlo simulations to verify the estimation performance of the proposed method. Using the same the geometry of distributed passive sensors in [11], the configuration is given in Table 1, shown in Figure 2, which contains *M* = 8 sensors (4 groups). The unit of the positions and velocities of sensors are meter (m) and meters per second (m/s), respectively. Firstly, Section 5.1 and Section 5.2 present the comparison of the CRLB with sensor location noise power and two types of positioning measurements noise power respectively. Secondly, Section 5.3 and Section 5.4 evaluate the performance of the proposed method by comparing with other localization estimators. In addition, as for moving or stationary source, the TDOAs and FDOAs are also given in the simulation both in near-field and far-field scenarios. The near-field and far-field moving source are located at ${\left[500,-500,600\right]}^{T}$ and ${\left[2000,-2500,3000\right]}^{T}$ with the same moving velocity ${\left[-30,-15,20\right]}^{T}$. The stationary near-field and far-field source position are ${\left[500,-500,600\right]}^{T}$ and ${\left[2000,-2500,3000\right]}^{T}$ respectively.

### 5.1. CRLB Comparison Versus Sensor Location Error

In this section, we compared the CRLB which has been derived in Section 4 with the CRLB in [11] illustrating the sensitivity to the sensor location errors of the CRLB. The TDOA and FDOA noise power are $\sigma_{t}^{2}=10^{-4}/c^{2}$ and $\sigma_{f}^{2}=10^{-5}/c^{2}$ respectively. $Q_{\alpha }$ is

(38) $$Q_{\alpha }={\left[\begin{array}{cc} Q_{t} & O\\ O & Q_{f} \end{array}\right]}_{M\times M}$$

where $Q_{t}$ is a (M/2)×(M/2) matrix with $c^{2}\sigma_{t}^{2}$ in the diagonal and $0.5c^{2}\sigma_{t}^{2}$ in all other elements [34] and $Q_{f}=Q_{t}\sigma_{f}^{2}/\sigma_{t}^{2}$. In addition, $Q_{\beta }$ is

(39) $$Q_{\beta }={\left[\begin{array}{cc} Q_{s} & O\\ O & {\dot{Q}}_{s} \end{array}\right]}_{6M\times 6M}$$

where $Q_{s}=\sigma_{s}^{2}I_{3M\times 3M}$, ${\dot{Q}}_{s}={\dot{\sigma }}_{s}^{2}I_{3M\times 3M}$, and ${\dot{\sigma }}_{s}^{2}=0.5\sigma_{s}^{2}$.

**Figure 3:**
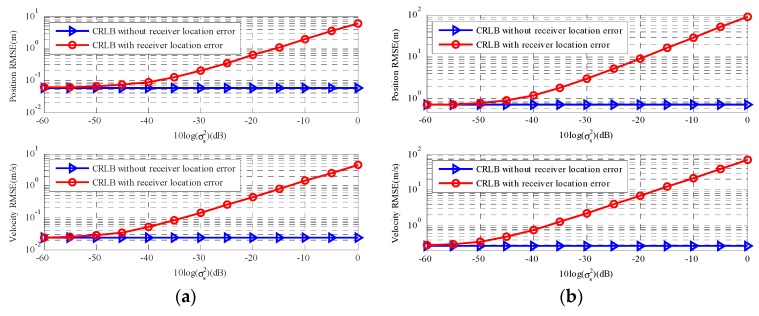
CRLB comparison versus sensor location error. (**a**) The near-field moving source; (**b**) The far-field moving source.

Figure 3 shows the trace of CRLB(u) and $CRLB\left(\dot{u}\right)$ versus sensor location error. With $\sigma_{s}^{2}$ increases, the gap between the two types of CRLB becomes more and more obvious and the larger the $\sigma_{s}^{2}$ is, the worse the estimation accuracy with sensor location uncertainties is. In Figure 3a, When the sensor location error power is $\sigma_{s}^{2}=10^{-3}\left(10\log\left(\sigma_{s}^{2}\right)=-30\mathrm{dB}\right)$, there are relative increases in CRLB for position u and velocity $\dot{u}$. Figure 3b exhibits the results for a far-field moving source and the observation is similar to Figure 3a. However, compared with a near-field source, the estimation error of far-field moving source clearly increases.

**Figure 4:**
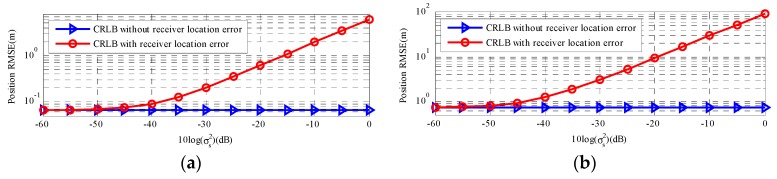
CRLB comparison versus sensor location error. (**a**) The near-field stationary source; (**b**) The far-field stationary source.

Figure 4 gives the results for two types of stationary sources. In this scenario, θ=u, α=r, β=s, $Q_{\alpha }=Q_{t}$, $Q_{\beta }=Q_{s}$ and the partial derivatives can be found in Appendix A and Appendix B. The results of two types of sources correspond with the moving source scenario and the CRLB of far-field stationary source changed more obvious than that of the near-field stationary source as the sensor location error increase. Based on the simulation results analyzed above, the sensor location geometry plays a significant role in the CRLB. If the accuracy of the sensor location coordinates cannot be guaranteed, it will have negative influence on estimated accuracy of source location, especially for far-field source scenarios. Thus, we should ensure the accuracy of the sensor location coordinates before we estimate the source location.

### 5.2. CRLB Comparison Versus Measurements Noise

In this section, we fix the sensor location error at $\sigma_{s}^{2}=10^{0}$, ${\dot{\sigma }}_{s}^{2}=0.5\times 10^{0}$ and present the comparisons of the two types of CRLBs which change with TDOA and FDOA noise power. This comparison will investigate the sensitivity to the TDOA and FDOA measurements noise of the CRLB. The true TDOA and FDOA values are added uncorrelated Gaussian noise with zero means.

**Figure 5:**
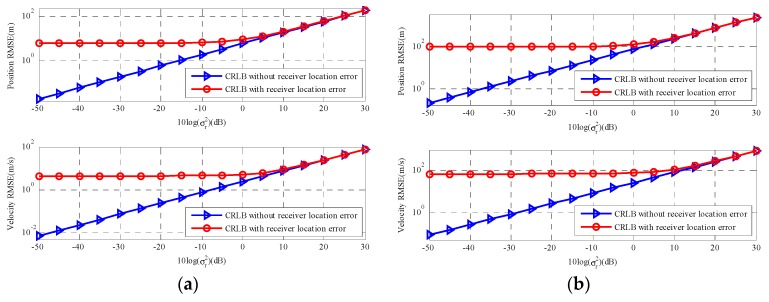
CRLB comparison versus measurements noise $\sigma_{r}$. (**a**) The near-field moving source; (**b**) The far-field moving source.

Figure 5 is the comparisons of the two types of CRLBs change with two types of positioning measurements noise power. As we can see, there is no remarkable change in the CRLB with sensor location error and the CRLB in [11] changed more dramatic than the CRLB with sensor location error when positioning measurements noise level is low. However, in Figure 5a, as the $\sigma_{r}^{2}$ increase (from 10^0.5^ to 10^3^), the two types of CRLBs exhibit the similar tendency and the CRLB with sensor location error presents a dramatic change. Figure 5b is the result for a far-field moving source. The observation is similar, and the two types of CRLBs for the far-field moving source are also higher than that for the far-field source.

**Figure 6:**
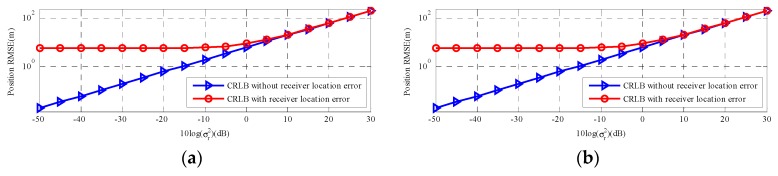
CRLB comparison versus measurements noise $\sigma_{r}$. (**a**) The near-field stationary source; (**b**) The far-field stationary source.

Figure 6 respectively gives the results for a near-field stationary source and a far-field stationary source, which has a similar tendency to moving source. In a word, according to Section 5.1 and Section 5.2, the accuracy of sensor location has a major impact on CRLB considering sensor location error far beyond positioning measurements noise. 

Overall, the CRLB with sensor location uncertainties is not sensitive to the positioning measurements noise power at low level. With this noise power increasing, the CRLB with sensor location uncertainties just increases slightly. Thus, the sensor location errors play a significant role in the source localization estimate and we should avoid these errors, which have a negative influence on estimated accuracy.

### 5.3. RMSE Comparison for A Near-Field Source

Section 5.3 uses numerical simulations to demonstrate the proposed method and to compare its performance with other localization estimators for a near-field source. Other simulation conditions are similar to Section 5.1. The estimation bias and accuracy are investigated for source as sensor location errors increase. The estimation accuracy is evaluated in terms of the RMSE, which is defined as $RMSE\left(u\right)=\sqrt{\left(1/K\right)\sum_{k=1}^{K}{\left\|u_{k}-u^{o}\right\|}^{2}}$ and $RMSE\left(\dot{u}\right)=\sqrt{\left(1/K\right)\sum_{k=1}^{K}{\left\|{\dot{u}}_{k}-{\dot{u}}^{o}\right\|}^{2}}$. The estimation bias in terms of norm of estimation bias is defined as $bias\left(u\right)=\left(1/K\right)\left\|\sum_{k=1}^{K}\left(u_{k}-u^{o}\right)\right\|$ and $bias\left(\dot{u}\right)=\left(1/K\right)\left\|\sum_{k=1}^{K}\left({\dot{u}}_{k}-{\dot{u}}^{o}\right)\right\|$, where $u^{o}$ and ${\dot{u}}^{o}$ stand for the true value of the source location, and each Monte Carlo estimate of source location is expressed as $u_{k}$ and ${\dot{u}}_{k}$. K=10000 is the number of independent Monte Carlo runs. In particular, we use the estimated and noisy measurement values to replace the true values in (31) throughout the simulations.

**Figure 7:**
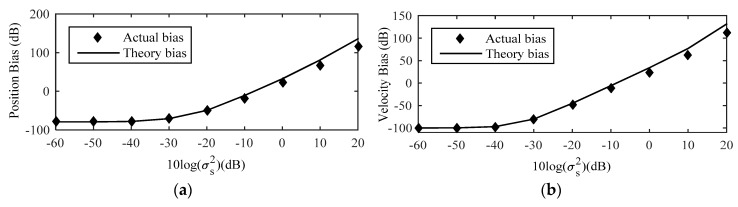
Comparison between theoretical and actual bias of near-field source location estimate by MLE. (**a**) Position Bias; (**b**) Velocity Bias.

Figure 7 shows the comparison between theoretical bias and actual bias of estimation of source location by MLE for a near-field source. As shown in this figure, the theoretical bias matches the actual bias well when the noise level is smaller than −10 dB. Therefore, it is efficient to use the theoretical bias to compensate the source location estimate and improve the estimated accuracy of the proposed method. However, with the increase of sensor location error noise level, the theoretical bias value gradually deviates from the actual bias, especially the source velocity bias. The major cause of this phenomenon is the approximation of the (14) such that the high-order terms are ignored during the process of derivation. Therefore, in order to obtain the more accurate estimation of source location and we should do the Taylor-series expansion of (14) at $\varphi^{o}$ up to high-order.

**Figure 8:**
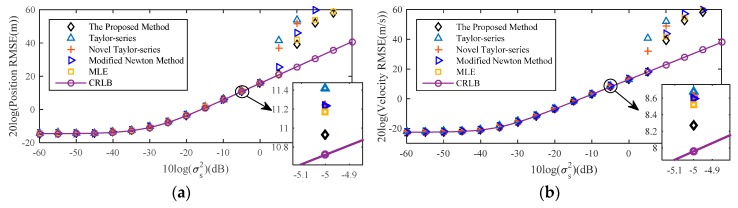
RMSE Comparison among different methods for the near-field source. (**a**) Position RMSE; (**b**) Velocity RMSE.

Figure 8 shows the RMSE of the proposed method with the sensor location error noise level increases, and comparing it with the existing localization algorithm Taylor-series method [14], the novel Taylor-series method [15], the modified Newton method [16], MLE [10], as well as CRLB considering sensor location uncertainties. The initial values of them are both true source location. The RMSE of proposed method is always higher than that of the other localization algorithms and all the algorithms can attain the CRLB at low moderate noise level. After compensating by (32), the position and velocity RMSE of the source decrease 3.16 dB and 2.36 dB respectively compared with the estimated result without considering the bias compensation when $\sigma_{s}^{2}\geq 10^{0}$. In the drawing of partial enlargement, compared with other localization algorithm, the RMSE of the proposed estimator is lower, which indicates that the proposed method exhibits the best performance.

**Figure 9:**
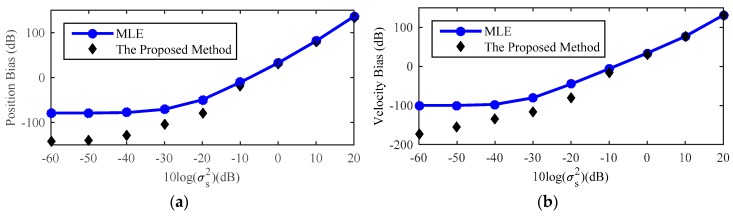
Bias comparison of the proposed method with the original MLE for near-field source. (**a**) Position Bias; (**b**) Velocity Bias.

In Figure 9, the estimation results clearly demonstrate that the bias of the proposed method is nonetheless smaller than the MLE for low sensor location error noise level. More specifically, when sensor location error noise is lower than −20 dB, the position and velocity bias of proposed method are at least 30 dB and 35 dB lower than the MLE. It is efficient to reduce the impact of the MLE bias on estimation. With the increase of the noise power, the original MLE is affected by the threshold effect, which leads to the decrease of the estimation performance.

### 5.4. RMSE Comparison for A Far-Field Source

**Figure 10:**
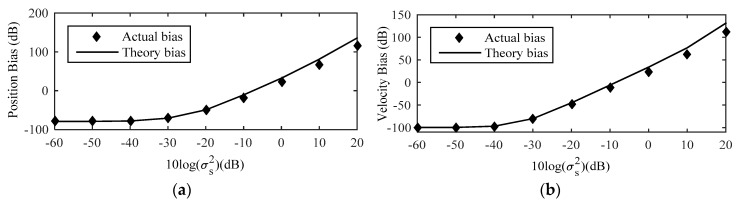
Comparison between theoretical and actual bias of far-field source location estimate by MLE. (**a**) Position Bias; (**b**) Velocity Bias.

This section is concerned with far-field source localization. Other simulation conditions are similar to Section 5.1. Figure 10 shows the comparison between theoretical and actual bias of estimation of source position and velocity by MLE. The trend of the result is the same as Figure 7 and compared with the result for the near-field source, the phenomenon that the theoretical bias value gradually deviates from the actual bias for the far-field source occurs earlier than that for the near-field source.

**Figure 11:**
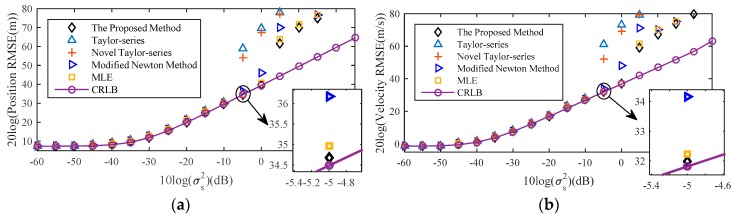
RMSE Comparison among different methods for the near-field source. (**a**) Position RMSE; (**b**) Velocity RMSE.

Figure 11 has similar simulation contents with the Figure 8, but the source is located in the far-field scenarios. As for far-field source, the distances between source and different sensors are approximately the same, hence the value of each range different is nearly equal to zero, which is indicated as the following

(40) $$\begin{array}{l} r_{1}^{o}\approx r_{2}^{o}\approx \cdots \approx r_{M}^{o}\\ r_{21}^{o}\approx r_{43}^{o}\approx \cdots \approx r_{M,M-1}^{o}\approx 0 \end{array}$$

Then, considering that the value of each range rate is far less than that of each range, the relationship between the range rate and the range can be approximately expressed as

(41) $$\frac{{\dot{r}}_{1}^{o}}{r_{1}^{o}}\approx \cdots \approx \frac{{\dot{r}}_{M}^{o}}{r_{M}^{o}}\approx 0$$

Thus, according to (40), (41) and the analysis mentioned above, the estimated performance for the far-field source is commonly worse than that for the near-field source. In this case, although the estimated performance for far-field source is not guaranteed when the noise level is high, the estimation precision of the proposed method is always higher than that of the other comparison algorithms, which is shown in partial enlargement. 

**Figure 12:**
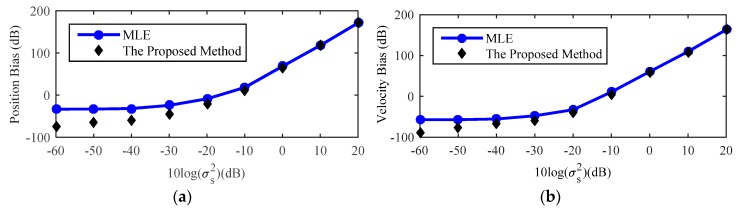
Bias comparison of the proposed method with the original MLE for far-field source. (**a**) Position Bias; (**b**) Velocity Bias.

Figure 12 is the result for a far-field source about the bias analysis of the proposed method and the trend of the result is the same as Figure 9. When compared with result of the near-field source in Figure 9, although the gap between the proposed method and MLE for the far-field source is smaller than that for the near-field source because of the two conditions shown in (41), the proposed method is still effective in reducing the bias of the MLE.

## 6. Conclusions

Moving source localization is a challenging problem due to its nonlinearity characteristic and increasing demand for high performance. To reduce estimation error and make the localization model closer to the practical environment, an algebraic moving source localization method using TDOA, FDOA and Doppler Rate measurements with receiver location errors is presented in this paper. The proposed method gives a final closed-form solution in the second step without iteration and extra mathematics operations by employing the basic idea of WLS processing. A new CRLB with receiver location errors is also derived. Theoretical analysis demonstrates that the proposed method can achieve CRLB under moderate noise conditions. Simulation results show that the proposed method can efficiently avoid the rank deficiency problem and outperforms the compared methods as the SNR and location error change.

## Figures and Tables

**Table 1:** Distributed Passive Sensors Configuration.

Groups	Sensor No.*i*	$x_{i}(m)$	$y_{i}(m)$	$z_{i}(m)$	${\dot{x}}_{i}(m/s)$	${\dot{y}}_{i}(m/s)$	${\dot{z}}_{i}(m/s)$
Group 1	1	−150	−600	200	10	20	−30
	2	50	−750	200	20	30	0
Group 2	3	500	−200	500	−10	0	10
	4	600	100	600	10	20	15
Group 3	5	100	600	800	−10	20	20
	6	−100	400	700	30	0	20
Group 4	7	−600	50	400	15	10	−15
	8	−750	−100	500	−20	−15	10

## Appendix A

The first derivatives of the α for φ is shown in this part. Based on the Section 2, let $\alpha ={\left[r^{T},{\dot{r}}^{T}\right]}^{T}$, $\beta ={\left[s^{T},{\dot{s}}^{T}\right]}^{T}$, $r={\left[r_{2,1},r_{4,3},\ldots ,r_{M,M-1}\right]}^{T}$, and $\dot{r}={\left[{\dot{r}}_{2,1},{\dot{r}}_{4,3},\ldots ,{\dot{r}}_{M,M-1}\right]}^{T}$. So, the $\partial \alpha /\partial \varphi^{T}$ in (18) can be expressed as

(A1) $$\frac{\partial \alpha }{\partial \varphi^{T}}={\left[\begin{array}{cc} \frac{\partial \alpha }{\partial \theta^{T}} & \frac{\partial \alpha }{\partial \beta^{T}} \end{array}\right]}_{M\times \left(6+6M\right)}$$

where

(A2) $$\frac{\partial \alpha }{\partial \theta^{T}}={\left[\begin{array}{cc} \frac{\partial r}{\partial u^{T}} & \frac{\partial r}{\partial {\dot{u}}^{T}}\\ \frac{\partial \dot{r}}{\partial u^{T}} & \frac{\partial \dot{r}}{\partial {\dot{u}}^{T}} \end{array}\right]}_{M\times 6},\;\frac{\partial \alpha }{\partial \beta^{T}}={\left[\begin{array}{cc} \frac{\partial r}{\partial s^{T}} & \frac{\partial r}{\partial {\dot{s}}^{T}}\\ \frac{\partial \dot{r}}{\partial s^{T}} & \frac{\partial \dot{r}}{\partial {\dot{s}}^{T}} \end{array}\right]}_{M\times 6M}$$

According to (5) and (7), we let

(A3) $$\begin{array}{l} x_{i}=r_{i}^{-1}{\left(u-s_{i}\right)}^{T}\\ v_{i}=r_{i}^{-1}{\left(\dot{u}-{\dot{s}}_{i}\right)}^{T}-r_{i}^{-2}{\dot{r}}_{i}{\left(u-s_{i}\right)}^{T} \end{array}$$

Thus, the partial derivatives of r and $\dot{r}$ with respect to u and $\dot{u}$ yields (A6), shown as

(A4) $$\frac{\partial r}{\partial u^{T}}=\frac{\partial \dot{r}}{\partial {\dot{u}}^{T}}={\left[\begin{array}{c} x_{2}-x_{1}\\ \vdots \\ x_{M}-x_{M-1} \end{array}\right]}_{\left(M/2\right)\times 3},\;\frac{\partial \dot{r}}{\partial u^{T}}={\left[\begin{array}{c} v_{2}-v_{1}\\ \vdots \\ v_{M}-v_{M-1} \end{array}\right]}_{\left(M/2\right)\times 3},\;\frac{\partial r}{\partial {\dot{u}}^{T}}=O_{\left(M/2\right)\times 3}$$

In addition, the partial derivatives of r and $\dot{r}$ with respect to s and $\dot{s}$ yields (A7), which are shown as

(A5) $$\begin{array}{l} \frac{\partial r}{\partial s^{T}}={\left[\begin{array}{ccccccc} x_{1} & -x_{2} & & & & & \\ & & x_{3} & -x_{4} & & & \\ & & & & \cdots & & \\ & & & & & x_{M-1} & -x_{M} \end{array}\right]}_{\left(M/2\right)\times 3M}\\ \frac{\partial \dot{r}}{\partial s^{T}}={\left[\begin{array}{ccccccc} v_{1} & -v_{2} & & & & & \\ & & v_{3} & -v_{4} & & & \\ & & & & \cdots & & \\ & & & & & v_{M-1} & -v_{M} \end{array}\right]}_{\left(M/2\right)\times 3M}\\ \frac{\partial r}{\partial {\dot{s}}^{T}}=O_{\left(M/2\right)\times 3M},\;\frac{\partial \dot{r}}{\partial {\dot{s}}^{T}}=\frac{\partial r}{\partial s^{T}} \end{array}$$

## Appendix B

The first derivatives of the β for φ is shown in this part. According to Appendix A, the $\partial \beta /\partial \varphi^{T}$ in (18) can be expressed as

(A6) $$\frac{\partial \beta }{\partial \varphi^{T}}={\left[\begin{array}{ccc} \frac{\partial s}{\partial \theta^{T}} & \frac{\partial s}{\partial s^{T}} & \frac{\partial s}{\partial {\dot{s}}^{T}}\\ \frac{\partial \dot{s}}{\partial \theta^{T}} & \frac{\partial \dot{s}}{\partial s^{T}} & \frac{\partial \dot{s}}{\partial {\dot{s}}^{T}} \end{array}\right]}_{6M\times \left(6+6M\right)}$$

where

(A7) $$\frac{\partial s}{\partial \theta_{1}^{T}}=\frac{\partial \dot{s}}{\partial \theta_{1}^{T}}=O_{3M\times 6},\;\frac{\partial \dot{s}}{\partial s^{T}}=\frac{\partial s}{\partial {\dot{s}}^{T}}=O_{3M\times 3M},\;\frac{\partial s}{\partial s^{T}}=\frac{\partial \dot{s}}{\partial {\dot{s}}^{T}}=I_{3M\times 3M}$$

## Appendix C

The second derivatives of the α for φ is shown in this part.

When the 1≤j≤M/2, the $\partial^{2}\alpha_{j}/\partial \varphi \partial \varphi^{T}$ in (18) can be expressed as

(A8) $$\frac{\partial^{2}\alpha_{j}}{\partial \varphi \partial \varphi^{T}}={\left[\begin{array}{cc} \frac{\partial^{2}r_{2j,2j-1}}{\partial \theta \partial \theta^{T}} & \frac{\partial^{2}r_{2j,2j-1}}{\partial \theta \partial \beta^{T}}\\ \frac{\partial^{2}r_{2j,2j-1}}{\partial \beta \partial \theta^{T}} & \frac{\partial^{2}r_{2j,2j-1}}{\partial \beta \partial \beta^{T}} \end{array}\right]}_{\left(6M+6\right)\times \left(6M+6\right)}$$

where

(A9) $$\begin{array}{cc} \frac{\partial^{2}r_{2j,2j-1}}{\partial \theta \partial \theta^{T}}={\left[\begin{array}{cc} \frac{\partial^{2}r_{2j,2j-1}}{\partial u\partial u^{T}} & \frac{\partial^{2}r_{2j,2j-1}}{\partial u\partial {\dot{u}}^{T}}\\ \frac{\partial^{2}r_{2j,2j-1}}{\partial \dot{u}\partial u^{T}} & \frac{\partial^{2}r_{2j,2j-1}}{\partial \dot{u}\partial {\dot{u}}^{T}} \end{array}\right]}_{6\times 6}, & \frac{\partial^{2}r_{2j,2j-1}}{\partial \theta \partial \beta^{T}}={\left[\begin{array}{cc} \frac{\partial^{2}r_{2j,2j-1}}{\partial u\partial s^{T}} & \frac{\partial^{2}r_{2j,2j-1}}{\partial u\partial {\dot{s}}^{T}}\\ \frac{\partial^{2}r_{2j,2j-1}}{\partial \dot{u}\partial s^{T}} & \frac{\partial^{2}r_{2j,2j-1}}{\partial \dot{u}\partial {\dot{s}}^{T}} \end{array}\right]}_{6\times 6M}\\ \frac{\partial^{2}r_{2j,2j-1}}{\partial \beta \partial \beta^{T}}={\left[\begin{array}{cc} \frac{\partial^{2}r_{2j,2j-1}}{\partial s\partial s^{T}} & \frac{\partial^{2}r_{2j,2j-1}}{\partial s\partial {\dot{s}}^{T}}\\ \frac{\partial^{2}r_{2j,2j-1}}{\partial \dot{s}\partial s^{T}} & \frac{\partial^{2}r_{2j,2j-1}}{\partial \dot{s}\partial {\dot{s}}^{T}} \end{array}\right]}_{6M\times 6M}, & \frac{\partial^{2}r_{2j,2j-1}}{\partial \beta \partial \theta^{T}}={\left(\frac{\partial^{2}r_{2j,2j-1}}{\partial \theta \partial \beta^{T}}\right)}^{T} \end{array}$$

According to (5) and (7), we let

(A10) $$X_{j}=\left(I_{3\times 3}-x_{j}^{T}x_{j}\right)r_{j}^{-1}$$

Therefore, the second-order partial derivatives of r with respect to θ yields (A13), shown as

(A11) $$\begin{array}{l} \frac{\partial^{2}r_{2j,2j-1}}{\partial u\partial u^{T}}=X_{2j}-X_{2j-1}\\ \frac{\partial^{2}r_{2j,2j-1}}{\partial \dot{u}\partial u^{T}}=\frac{\partial^{2}r_{2j,2j-1}}{\partial u\partial {\dot{u}}^{T}}=\frac{\partial^{2}r_{2j,2j-1}}{\partial \dot{u}\partial {\dot{u}}^{T}}=O_{3\times 3} \end{array}$$

In addition, the second-order partial derivatives of r with respect to θ and β yields (A14), which are shown as

(A12) $$\begin{array}{l} \frac{\partial^{2}r_{2j,2j-1}}{\partial u\partial s_{k}^{T}}=\{\begin{array}{cc} X_{j} & k=2j-1\\ -X_{j} & k=2j\\ O_{3\times 3} & else \end{array}\\ \frac{\partial^{2}r_{2j,2j-1}}{\partial u\partial {\dot{s}}^{T}}=\frac{\partial^{2}r_{2j,2j-1}}{\partial \dot{u}\partial s^{T}}=\frac{\partial^{2}r_{2j,2j-1}}{\partial \dot{u}\partial {\dot{s}}^{T}}=O_{3\times 3M} \end{array}$$

The second-order partial derivatives of r with respect to β are expressed as

(A13) $$\begin{array}{l} \mathrm{When}\;k_{1}=k_{2}\\ \frac{\partial^{2}r_{2j,2j-1}}{\partial s_{k_{1}}\partial s_{k_{2}}^{T}}=\{\begin{array}{cc} -X_{j} & k_{1}=k_{2}=2j-1\\ X_{j} & k_{1}=k_{2}=2j\\ O_{3\times 3} & else \end{array}\\ \mathrm{When}\;k_{1}\neq k_{2}\\ \frac{\partial^{2}r_{2j,2j-1}}{\partial s_{k_{1}}\partial s_{k_{2}}^{T}}=O_{3\times 3} \end{array}$$

and

(A14) $$\frac{\partial^{2}r_{2j,2j-1}}{\partial s\partial {\dot{s}}^{T}}=\frac{\partial^{2}r_{2j,2j-1}}{\partial \dot{s}\partial s^{T}}=\frac{\partial^{2}r_{2j,2j-1}}{\partial \dot{s}\partial {\dot{s}}^{T}}=O_{3M\times 3M}$$

When M/2+1≤j≤M, the $\partial^{2}\alpha_{j}/\partial \varphi \partial \varphi^{T}$ in (18) can be expressed as

(A15) $$\frac{\partial^{2}\alpha_{j}}{\partial \varphi \partial \varphi^{T}}={\left[\begin{array}{cc} \frac{\partial^{2}{\dot{r}}_{2j-M,2j-1-M}}{\partial \theta \partial \theta^{T}} & \frac{\partial^{2}{\dot{r}}_{2j-M,2j-1-M}}{\partial \theta \partial \beta^{T}}\\ \frac{\partial^{2}{\dot{r}}_{2j-M,2j-1-M}}{\partial \beta \partial \theta^{T}} & \frac{\partial^{2}{\dot{r}}_{2j-M,2j-1-M}}{\partial \beta \partial \beta^{T}} \end{array}\right]}_{\left(6M+6\right)\times \left(6M+6\right)}$$

where

(A16) $$\begin{array}{l} \frac{\partial^{2}{\dot{r}}_{2j-M,2j-1-M}}{\partial \theta \partial \theta^{T}}={\left[\begin{array}{cc} \frac{\partial^{2}{\dot{r}}_{2j-M,2j-1-M}}{\partial u\partial u^{T}} & \frac{\partial^{2}{\dot{r}}_{2j-M,2j-1-M}}{\partial u\partial {\dot{u}}^{T}}\\ \frac{\partial^{2}{\dot{r}}_{2j-M,2j-1-M}}{\partial \dot{u}\partial u^{T}} & \frac{\partial^{2}{\dot{r}}_{2j-M,2j-1-M}}{\partial \dot{u}\partial {\dot{u}}^{T}} \end{array}\right]}_{6\times 6}\\ \frac{\partial^{2}{\dot{r}}_{2j-M,2j-1-M}}{\partial \theta \partial \beta^{T}}={\left[\begin{array}{cc} \frac{\partial^{2}{\dot{r}}_{2j-M,2j-1-M}}{\partial u\partial s^{T}} & \frac{\partial^{2}{\dot{r}}_{2j-M,2j-1-M}}{\partial u\partial {\dot{s}}^{T}}\\ \frac{\partial^{2}{\dot{r}}_{2j-M,2j-1-M}}{\partial \dot{u}\partial s^{T}} & \frac{\partial^{2}{\dot{r}}_{2j-M,2j-1-M}}{\partial \dot{u}\partial {\dot{s}}^{T}} \end{array}\right]}_{6\times 6M}\\ \frac{\partial^{2}{\dot{r}}_{2j-M,2j-1-M}}{\partial \beta \partial \beta^{T}}={\left[\begin{array}{cc} \frac{\partial^{2}{\dot{r}}_{2j-M,2j-1-M}}{\partial s\partial s^{T}} & \frac{\partial^{2}{\dot{r}}_{2j-M,2j-1-M}}{\partial s\partial {\dot{s}}^{T}}\\ \frac{\partial^{2}{\dot{r}}_{2j-M,2j-1-M}}{\partial \dot{s}\partial s^{T}} & \frac{\partial^{2}{\dot{r}}_{2j-M,2j-1-M}}{\partial \dot{s}\partial {\dot{s}}^{T}} \end{array}\right]}_{6M\times 6M}\\ \frac{\partial^{2}{\dot{r}}_{2j-M,2j-1-M}}{\partial \beta \partial \theta^{T}}={\left(\frac{\partial^{2}{\dot{r}}_{2j-M,2j-1-M}}{\partial \theta \partial \beta^{T}}\right)}^{T} \end{array}$$

Let

(A17) $$\begin{array}{l} Y_{j}={\dot{r}}_{j}r_{j}^{-2}\left(3x_{j}^{T}x_{j}-I_{3\times 3}\right)\\ w_{j}=r_{j}^{-1}{\left(\dot{u}-{\dot{s}}_{j}\right)}^{T}\\ W_{j}=r_{j}^{-1}x_{j}^{T}w_{j}\\ \Psi_{j}=Y_{j}-W_{j}-W_{j}^{T} \end{array}$$

The second-order partial derivatives of $\dot{r}$ with respect to θ yields (A20), shown as

(A18) $$\begin{array}{l} \frac{\partial^{2}{\dot{r}}_{2j-M,2j-1-M}}{\partial u\partial u^{T}}=\Psi_{2j-M}-\Psi_{2j-1-M},\;\frac{\partial^{2}{\dot{r}}_{2j-M,2j-1-M}}{\partial \dot{u}\partial u^{T}}=X_{2j-M}-X_{2j-1-M}\\ \frac{\partial^{2}{\dot{r}}_{2j-M,2j-1-M}}{\partial u\partial {\dot{u}}^{T}}=X_{2j-M}-X_{2j-1-M},\;\frac{\partial^{2}{\dot{r}}_{2j-M,2j-1-M}}{\partial \dot{u}\partial {\dot{u}}^{T}}=O_{3\times 3} \end{array}$$

In addition, the second-order partial derivatives of $\dot{r}$ with respect to θ and β yields (A21), which are shown as

(A19) $$\begin{array}{l} \frac{\partial^{2}{\dot{r}}_{2j-M,2j-1-M}}{\partial u\partial s_{k}^{T}}=\{\begin{array}{cc} \Psi_{2j-M} & k=2j-1\\ -\Psi_{2j-M} & k=2j\\ O_{3\times 3} & else \end{array}\\ \frac{\partial^{2}{\dot{r}}_{2j-M,2j-1-M}}{\partial u\partial {\dot{s}}_{k}{}^{T}}=\frac{\partial^{2}r_{2j,2j-1}}{\partial u\partial s_{k}^{T}},\;\frac{\partial^{2}{\dot{r}}_{2j-M,2j-1-M}}{\partial \dot{u}\partial s^{T}}=\frac{\partial^{2}r_{2j,2j-1}}{\partial u\partial s^{T}},\;\frac{\partial^{2}{\dot{r}}_{2j-M,2j-1-M}}{\partial \dot{u}\partial {\dot{s}}^{T}}=O_{3M\times 3M} \end{array}$$

The second-order partial derivatives of $\dot{r}$ with respect to β are expressed as

(A20) $$\begin{array}{l} \mathrm{When}\;k_{1}=k_{2}\\ \frac{\partial^{2}{\dot{r}}_{2j-M,2j-1-M}}{\partial s_{k_{1}}\partial s_{k_{2}}^{T}}=\{\begin{array}{cc} -\Psi_{2j-M} & k_{1}=k_{2}=2j-1\\ \Psi_{2j-M} & k_{1}=k_{2}=2j\\ O_{3\times 3} & else \end{array}\\ \mathrm{When}\;k_{1}\neq k_{2}\\ \frac{\partial^{2}{\dot{r}}_{2j-M,2j-1-M}}{\partial s_{k_{1}}\partial s_{k_{2}}^{T}}=O_{3\times 3} \end{array}$$

and

(A21) $$\frac{\partial^{2}{\dot{r}}_{2j-M,2j-1-M}}{\partial s\partial {\dot{s}}^{T}}=\frac{\partial^{2}{\dot{r}}_{2j-M,2j-1-M}}{\partial \dot{s}\partial s^{T}}=\frac{\partial^{2}r_{2j,2j-1}}{\partial u\partial s^{T}},\;\frac{\partial^{2}{\dot{r}}_{2j-M,2j-1-M}}{\partial \dot{s}\partial {\dot{s}}^{T}}=O_{3\times 3}$$

## Appendix D

The second derivatives of the β for φ is shown as following:

When 1≤j≤M/2, the $\partial^{2}\beta_{j}/\partial \varphi \partial \varphi^{T}$ in (18) can be expressed as

(A22) $$\frac{\partial^{2}\beta_{j}}{\partial \varphi \partial \varphi^{T}}=O_{\left(6M+6\right)\times \left(6M+6\right)}$$

When M/2+1≤j≤M, the $\partial^{2}\beta_{j}/\partial \varphi \partial \varphi^{T}$ in (18) can be expressed as

(A23) $$\frac{\partial^{2}\beta_{2j-M,2j-1-M}}{\partial \varphi \partial \varphi^{T}}=O_{\left(6M+6\right)\times \left(6M+6\right)}$$
